# Innovation and behavioral flexibility in wild redfronted lemurs (*Eulemur rufifrons*)

**DOI:** 10.1007/s10071-015-0844-6

**Published:** 2015-02-12

**Authors:** Franziska Huebner, Claudia Fichtel

**Affiliations:** Behavioral Ecology and Sociobiology Unit, German Primate Center, Kellnerweg 4, 37077 Göttingen, Germany

**Keywords:** Innovation, Problem solving, Prior knowledge, Behavioral flexibility, Persistence, Primates

## Abstract

Innovations and problem-solving abilities can provide animals with important ecological advantages as they allow individuals to deal with novel social and ecological challenges. Innovation is a solution to a novel problem or a novel solution to an old problem, with the latter being especially difficult. Finding a new solution to an old problem requires individuals to inhibit previously applied solutions to invent new strategies and to behave flexibly. We examined the role of experience on cognitive flexibility to innovate and to find new problem-solving solutions with an artificial feeding task in wild redfronted lemurs (*Eulemur rufifrons*). Four groups of lemurs were tested with feeding boxes, each offering three different techniques to extract food, with only one technique being available at a time. After the subjects learned a technique, this solution was no longer successful and subjects had to invent a new technique. For the first transition between task 1 and 2, subjects had to rely on their experience of the previous technique to solve task 2. For the second transition, subjects had to inhibit the previously learned technique to learn the new task 3. Tasks 1 and 2 were solved by most subjects, whereas task 3 was solved by only a few subjects. In this task, besides behavioral flexibility, especially persistence, i.e., constant trying, was important for individual success during innovation. Thus, wild strepsirrhine primates are able to innovate flexibly, suggesting a general ecological relevance of behavioral flexibility and persistence during innovation and problem solving across all primates.

**Electronic supplementary material:**

The online version of this article (doi:10.1007/s10071-015-0844-6) contains supplementary material, which is available to authorized users.

## Introduction

 The ability to innovate and to find new problem-solving strategies can have important fitness consequences for animals. More innovative individuals or species enhance their ability to exploit new resources, or to use existing resources more efficiently. They may even invade or create new niches or survive the invasion of another species (Kummer and Goodall 1985; Laland et al. 1996; Reader and Laland 2003; Sol et al. 2005; Ramsey et al. 2007; Morand-Ferron and Quinn 2011). For example, innovative anti-predator responses against novel predators (Berger et al. 2001) and adjusted breeding behaviors help animals to survive in changing ecological conditions (Brooke et al. 1998).

Animal innovation has been defined as “a solution to a novel problem or a novel solution to an old problem” (Kummer and Goodall 1985, p. 205). Innovation can also be considered as a process that results in a new or modified learned behavior, leading to the introduction of novel behavioral variants into the behavioral repertoire of a population (Reader and Laland 2003). Until today, research on innovation and problem solving has mainly focused on analyzing anecdotal accounts from the literature (e.g., Nicolakakis et al. 2003; Reader and Laland 2003), or innovations were elicited by presenting novel problems to captive animals (e.g., Köhler 1925; Visalberghi et al. 1995; Heinrich and Bugnyar 2005; Bond et al. 2007; Liker and Bókony 2009; Manrique et al. 2013).

Observational studies of innovations in the field are rare, as innovations are scarce and unpredictable (e.g., Gajdon et al. 2006; Turner et al. 2012; Schnoell and Fichtel 2013). Moreover, in order to recognize a behavior as an innovation, long-term behavioral observations are required, complicating field studies even more (van Schaik et al. 2006). However, a few studies have successfully implemented an experimental approach to study innovations in animals in their natural environment (birds: Webster and Lefebvre 2001; Bouchard et al. 2007; Boogert et al. 2010; Morand-Ferron and Quinn 2011; Morand-Ferron et al. 2011; mammals: Biro et al. 2003; Benson-Amram and Holekamp 2012; Thornton and Samson 2012). In contrast to studies on captive animals, which mainly tested separated animals with novel problem-solving tasks (e.g., Manrique et al. 2013), field experiments have the potential to provide more insights into the factors that drive innovation in nature as an entire free-ranging social group can be tested (Ramsey et al. 2007; Reader and Biro 2010).

Recent research revealed that various factors influence problem-solving abilities and behavioral plasticity (Kappeler et al. 2013; Snell-Rood 2013). Innovation rates were found to be a useful tool to quantify species differences in cognition and behavioral flexibility in birds (Lefebvre et al. 1997, 1998; Lefebvre 2000) and primates (Reader and Laland 2002). These studies revealed that innovation rates correlate with relative brain size in both taxa, with more innovative species having enlarged associative brain areas (Lefebvre et al. 1997, 2004; Timmermans et al. 2000; Reader and Laland 2002). Perceptual and learning differences may also influence innovation rates, with individuals that are able to perceive the causal structure of a problem or to generalize across different problems being more likely to innovate (Day et al. 2003).

Also, within-species differences in innovation rates exist. Various factors such as sex, age and social status (Reader and Laland 2001) and also individual characteristics such as personality and internal states play an important role in innovation and learning of new problem-solving strategies (Lefebvre 2000; Lewis 2002; Reader et al. 2011, reviewed in Brosnan and Hopper 2014). For example, exploration and novelty responses as well as constant trying are important behavioral processes during innovation in a range of species (e.g., Laland and Reader 1999; Webster and Lefebvre 2001; Day et al. 2003; Greenberg 2003; Tebbich et al. 2009, 2010; Cole et al. 2011; Thornton and Samson 2012; Benson-Amram and Holekamp 2012).

Finally, prior knowledge plays an important role in an animal’s ability to innovate and to solve problems (e.g., Köhler 1925; Epstein et al. 1984; Manrique et al. 2013). Prior experience with objects and their structural propensities can facilitate problem solving (e.g., Birch 1945), and already shaped behaviors can lead to novel solutions by an automatic chaining process (Epstein et al. 1984; Epstein 1987). However, in “finding a novel solution to an old problem” cases of innovation, prior knowledge might also hinder an animal to innovate. Here, prior knowledge could produce mental blockages, like functional fixedness, when objects like tools have fixed functions gained by past experience, which in turn hinders novel usage (Duncker and Lees 1945; Hanus et al. 2011). Before a novel solution can be found, old, previously learned solutions have to be inhibited, making these kinds of tasks particularly difficult (Manrique et al. 2013). Several studies on great apes have reported this form of conservatism, i.e., animals have problems or are reluctant to explore alternative solutions and techniques after having successfully mastered a particular technique or solution (e.g., Marshall-Pescini and Whiten 2008; Gruber et al. 2011; Hanus et al. 2011). For example, chimpanzees (*Pan troglodytes*) that had become proficient with a specific technique to acquire food were reluctant to switch to an alternative technique, even though they knew that the other technique was available and more efficient (Hrubesch et al. 2009). Three subjects even stayed with their learned and specialized technique after it was made ineffective, demonstrating pronounced conservatism (Hrubesch et al. 2009).

Similarly, keas (*Nestor notabilis*) and New Caledonian crows (*Corvus moneduloides*) were confronted with a multi-access feeding box, containing four different techniques to extract food (Auersperg et al. 2011). Once a subject had learned a specific technique, this technique was blocked and it had to abandon the old solution and to learn a new solution to the same problem. Only one subject of each species demonstrated such high behavioral flexibility and mastered all four tasks. Great apes confronted with a similar food extraction task, for which they had to learn different solutions in subsequent trials, were able to adjust their behavior flexibly and showed high degrees of inhibitory control during innovation (Manrique et al. 2013). Only orangutans (*Pongo abelli*) did not solve the third task.

However, studies of free-ranging animals focusing on innovation and behavioral flexibility during multiple problem solving are still missing. Moreover, studies on innovation in captive animals may suffer from low external validity because by-products of a captive lifestyle, for example reduced neophobia toward human objects, may influence innovation rates (Webster and Lefebvre 2001; Ramsey et al. 2007). Interestingly, comparisons between captive and wild animals of the same species found that captive animals had better technical problem-solving abilities, resulting in higher innovation rates (birds: Webster and Lefebvre 2001; Gajdon et al. 2004; Bouchard et al. 2007; hyenas: Benson-Amram et al. 2012). Therefore, observing innovation rates and problem-solving abilities as well as testing associated behavioral flexibility in wild animals can help understand the importance of innovations in a species’ natural habitat.

In this study, we tested behavioral flexibility to innovate and to find new problem-solving techniques with an artificial feeding task in wild redfronted lemurs (*Eulemur rufifrons*). Lemurs are interesting subjects for understanding the evolution of primate cognition for several reasons. First, lemurs and other strepsirrhine primates are phylogenetically the most basal living primates (Fichtel and Kappeler 2010). Furthermore, lemurs innovate in the wild [e.g., ringtailed lemurs (*Lemur catta*): Kendal et al. 2010, redfronted lemurs: Schnoell and Fichtel 2012, 2013]. Moreover, studies with captive brown and black lemurs (*Eulemur fulvus* and *Eulemur macaco*) showed that they are in principle capable of self-control (Genty et al. 2004; Glady et al. 2012). Thus, lemurs exhibit the necessary cognitive abilities (innovation and inhibitory control) required for this study.

Redfronted lemurs were able to solve a two-option feeding box task (Schnoell and Fichtel 2012) and spontaneously innovated a new foraging technique in the wild (Schnoell and Fichtel 2013). Furthermore, a recent social diffusion experiment with the same groups of redfronted lemurs participating in the present study examined long-term behavioral preferences for one of the two possible techniques to open a feeding box (Schnoell et al. 2014). Some individuals developed a stable preference for one technique over at least two consecutive years, which is indicative of conservative behavior. However, other subjects switched between having a preference for one technique or no preference (Schnoell et al. 2014). These findings indicate that individual differences in the degree of conservatism and behavioral flexibility exist when subjects can freely choose between techniques and when both techniques are equally difficult and rewarding. Thus, the aim of this study was to test the role of prior knowledge on lemurs’ problem-solving abilities and innovativeness.

Specifically, we tested whether lemurs are also able to learn a new food extracting technique after they learned to apply a previous, now obsolete technique efficiently. To this end, redfronted lemurs were provided with a feeding box offering three different techniques to open it and to extract a reward. For the first transition between techniques, subjects had to extend the previously learned solution; therefore, we predicted that the previously acquired knowledge and experience with the task would facilitate subjects’ learning. In contrast, during the second transition from task 2–3, prior knowledge was not helpful but supposedly hindering as now the previous technique was unsuccessful as animals were presented with a totally new problem. Thus, subjects had to inhibit the previously successful solution in order to learn a new, more difficult technique, requiring cognitive flexibility and inhibitory control during innovation and problem solving.

## Methods

### Study site and subjects

The study was conducted at the research station of the German Primate Center in Kirindy Forest, a dry deciduous forest located about 60 km north of Morondava in Western Madagascar (Kappeler and Fichtel 2012a). The study site is managed within a 12,500 ha forestry concession operated by the Centre National de Formation, d’Etude et de Recherche en Environnement et Foresterie (CNFEREF), Morondava. Data collection proceeded at the beginning of the dry season from mid-May until mid-August 2013. The experiments were conducted with four groups of redfronted lemurs (group A, B, F, J), ranging in size from 5 to 10 individuals. In total, 29 individuals were tested in the experiments: 23 of which interacted with the boxes in all three experimental tasks, three individuals participated only in one or two tasks, one individual disappeared after participating in task 2, and two individuals never interacted with the boxes. All subjects were well habituated to the presence of humans and individually marked with combinations of nylon collars and pendants or radio collars. Due to previous studies on social learning (Schnoell and Fichtel 2012; Schnoell et al. 2014), lemurs were familiar with the general experimental procedure, i.e., artificial feeding boxes, and highly motivated to extract food rewards, but naïve to the specific experimental apparatuses presented in this study.

### Apparatus

**Fig. 1:**
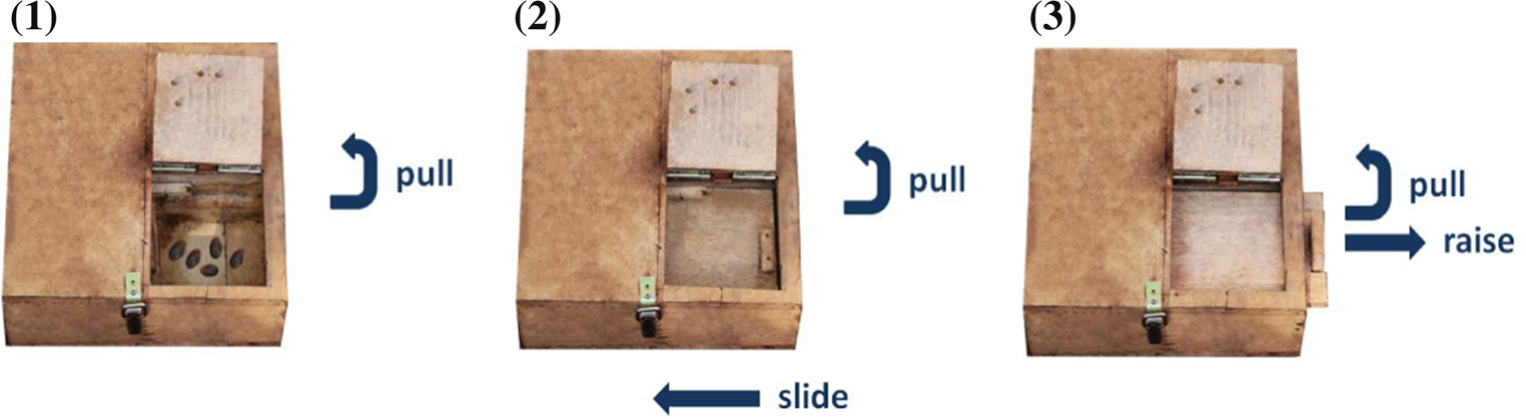
Feeding boxes with the respective techniques for the three tasks. **1** Task 1: pull technique, **2** task 2: pull-slide technique and **3** task 3: pull-raise technique

The experimental apparatus consisted of a wooden feeding box (measures 28 × 28 × 10 cm) offering three different techniques to obtain a reward with only one technique available at a time (Fig. 1). Boxes were baited with raisins and pieces of oranges, which subjects could only smell before opening. Techniques in the three test conditions differed in difficulty and required gradually more demanding manipulative tactics to extract the rewards.

In all three tasks, subjects needed to open a lid to reach the food reward via the same opening in the box (measures 10 × 10 cm). In task 1, subjects had to open a covering lid by pulling it (pull technique; see video Online Resource 1). The lid was constructed in such a way that animals had to hold the lid so that it did not fall back. In this way, boxes were available for multiple opening trials without necessarily being baited again and scrounging by other animals was hampered. Task 2 (pull-slide technique) required the use of both hands, one hand to pull the lid open (like in task 1), the other to slide an extra wooden board to the left side (see video Online Resource 2). For task 3 (pull-raise technique), the subjects again needed to pull the lid open, but now an extra wooden board had to be raised to the right with the other hand from outside of the box opening (see video Online Resource 3). This technique was more difficult to learn via trial-and-error than the other techniques because subjects could not simply use the smell of the reward as a cue for manipulation. As only one female subject succeeded in this task (in the following named T3a), we slightly modified it after the first 10 sessions. Now (T3b), the lid remained open after a subject successfully pulled it open, and subjects could use both hands to raise the board to the right.

### Procedure

Each group was tested with six feeding boxes simultaneously in order to prevent monopolization of boxes by a few animals as this could often be observed when a whole group of lemurs was tested (e.g., Fornasieri et al. 1990; Anderson et al. 1992; Schnoell and Fichtel 2012). The baiting of boxes took place out of sight of the lemurs to avoid the association of humans with food. Boxes were placed in open areas in the forest before the respective group was attracted with a clicker noise. Testing took place in the morning between 8:00 and 11:00 a.m. and in the afternoon between 01:30 and 5:00 p.m. Each group was tested once or twice per day in a randomized order with at least 3 h between experiments with the same group. A session began when the first subject entered the 7-m radius around the boxes and ended when subjects did not contact the boxes for 4 min or the last subject left the 7-m radius. During a session, we performed scans every second minute. In order to control for influences of social learning, positions of all individuals within a 7 m radius of the boxes were noted as well as whether they observed other subjects opening boxes, i.e., the subject’s head was turned toward another individual manipulating a box.

At the beginning of data collection, the boxes could be opened with the pull technique. After 20 sessions, the boxes were changed so that the pull-slide technique was necessary to obtain the reward for the next 20 sessions with each group. Subsequently, the pull-raise technique was tested for 30 sessions in total, 10 sessions with task 3a and another 20 sessions with task 3b.

### Data scoring and analyses

All test sessions were video-taped from different angles with two video cameras. We determined the number of successful trials, i.e., successful opening of the box with the respective technique, for all subjects in the three test conditions. Moreover, individuals’ contacts with the boxes and the different kinds of unsuccessful task manipulations were counted. To compare the general participation of subjects in the different tasks and in the course of sessions within a task, we conducted a GLM with task and session as fixed factors. We classified subjects as juveniles (up to 2.5 years) or adults (more than 2.5 years) (Kappeler and Fichtel 2012b). To test for sex and age effects on performance, we conducted proportion tests. To control for the potential influence of social learning in successful subjects, we divided the number of scans a subject observed others manipulating the box by the total number of scans of an individual. To determine whether this rate of observing others influenced the number of trials a subject needed until the first success, we conducted a Spearman’s rank correlation.

To determine whether the proportion of successful subjects differed between the test conditions, i.e., between the different tasks, we conducted a Cochran *Q* test for all tasks and a McNemar test for pairwise comparisons. For analyzing how fast subjects learned a technique, we recorded for each task the number of unsuccessful trials (manipulation of the box with nose or hands) until a subject successfully opened the feeding box for the first time. The efficiency of each successful individual in retrieving a reward was calculated by dividing the number of successful trials by the number of total trials manipulating functional parts of the boxes in a given test condition. For the analysis of the unsuccessful trials until a subject’s first success and the efficiency of subjects, we conducted Friedman rank sum tests for successful individuals in all three test conditions and pairwise comparisons with a signed Wilcoxon matched pairs test.

For the analysis of the transition between tasks and techniques, we noted for every unsuccessful trial the specific technique that was applied. In this way, it was possible to investigate for how many trials and sessions subjects continued to use the previously learned technique after test conditions changed. In task 2, this previous technique was pulling the first lid open without further manipulation of the box. In task 3, subjects’ attempts to slide the wooden board to the left were counted as previous technique. For task 3, the number of trials with the previous technique (T2) in Task 3a and Task 3b was added up and square root transformed to calculate a generalized linear model (GLM) with task and session as fixed factors. To compare the number of these “previous technique attempts” in task 3 between successful and unsuccessful subjects that had learned to open the boxes in task 1 and task 2, and to compare the total number of unsuccessful trials in task 3 in these subjects, we performed Mann–Whitney *U* tests.

To analyze the explorative behavior of subjects, we calculated an exploration diversity (ED) score for each subject in each task. For these scores, we counted the number of different behaviors subjects exhibited when interacting with the boxes. For task 1, up to three different behaviors were observed: contacting the boxes, manipulating nonfunctional parts of the boxes, and pulling the lid. For task 2, the same three behaviors were observed as well as manipulating the wooden board as an additional behavior resulting in the highest possible ED score of 4. In task 3, additively manipulating the outer aspect of the wooden board (that was outside of the box opening; see Fig. 1) was counted, resulting in five possible behaviors for this task. For the comparison of ED scores between successful and unsuccessful subjects in each task, we conducted a Mann–Whitney *U* test. We compared ED scores between tasks by using a Friedman rank sum test with additional pairwise comparisons via Wilcoxon matched pairs test. For all three tasks, successful task manipulations in 10 % of test sessions were independently scored by a second observer (total *N* of 28 sessions, randomly selected) to assess inter-observer reliability, which was excellent (intra-class correlation coefficient = 0.98). Statistical tests were conducted using R 2.15.1 and SPSS.

## Results

### General participation and effects of subjects’ age and sex on innovation success

**Fig. 2:**
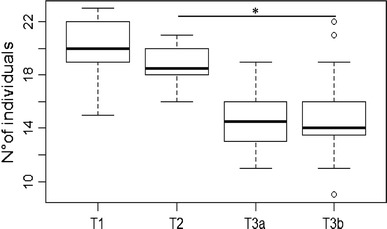
Number of individuals that contacted the boxes for all sessions in each task. Represented are median (*black bars*), interquartile range (*boxes*), upper and lower hinge (*whiskers*) and outliers (*circles*). *Asterisks* indicate significant differences with *P < 0.05

In total, 27 out of 29 individuals of the four groups contacted the feeding boxes at least once; two subjects were repeatedly in sight of the boxes but never touched them. On average, progressively fewer individuals contacted the feeding boxes from task 1 to task 3b, with significantly fewer individuals contacting the boxes in task 3b compared with 2 (GLM, *df* = 5, *P* < 0.05; Fig. 2; Table 1). Session in general had no influence on the number of subjects contacting the feeding boxes, but there was a significant interaction of task 1 with session; more individuals contacted the boxes over the course of the 20 sessions of task 1. Only adult individuals solved the tasks and successfully opened the feeding boxes, whereas the seven juveniles contacted the boxes but failed to open them [Proportion test, *N* (juveniles) = 7, *N* (adults) = 22, *χ* ^2^ = 7.344, *P* = 0.007]. Females tended to be more likely to successfully open the boxes, but this effect was not statistically significant (Table 2).

**Table 1:** Parameter estimation for the generalized linear model (GLM) on the number of individuals contacting the boxes in each task and number of trials with previous technique in each session in task 3

Fixed factors	Estimate	SE	*P* value
Number of individuals contacting the boxes in each task			
Intercept (Task 3b)	16.12	1.08	<0.001
Task 1	2.23	1.54	0.15
Task 2	3.74	1.54	<0.05
Task 3a	−1.39	1.93	0.48
Session	−0.12	1.93	0.19
Task 1: session	0.27	0.13	<0.05
Task 2: session	0.02	0.13	0.91
Task 3a: session	0.11	0.27	0.67
Number of trials with previous technique in task 3			
Intercept (Task 3a)	6.17	0.53	<0.001
Task 3b	−3.59	0.64	<0.001
Session	−0.56	0.09	<0.001
Task 3b: session	0.41	0.09	<0.001

**Table 2:** Number of successful female and male subjects for the different tasks (T)

	*N* successful females	*N* unsuccessful females	*N* successful males	*N* unsuccessful males	*P* proportion test
T1	9	7	6	7	0.867
T2	9	7	5	8	0.562
T3b	6	10	1	11	0.186

### Social learning control

The rate of observing other individuals manipulating the box until a subject’s first success was not correlated with the number of unsuccessful trials an animal made until its first success in all three tasks (Spearman’s rank correlation: T1: *N* = 15, *ρ* = 0.061, *P* = 0.828; T2: *N* = 14, *ρ* = 0.019, *P* = 0.949; T3b: *N* = 7, *ρ* = 0.427, *P* = 0.399). Therefore, independent of test condition, subjects that observed other conspecifics opening the box were not faster in learning a technique.

### Performance in the different tasks

In task 1, 15 subjects successfully opened the boxes [mean number of successful trials = 126.8 ± 110.49 (SD); Online Resource 5], and subjects succeeded after 1.53 ± 2 (mean ± SD) unsuccessful attempts. Task 2 was successfully solved by 14 individuals (mean number of successful trials = 158.571 ± 116.27); only one individual that solved task 1 was not successful in task 2 (for a general overview of the performance of subjects see Online Resource 5). Subjects tried on average 3.43 ± 4.55 times unsuccessfully to manipulate the boxes before their first success. In task 3a, only one subject successfully opened the boxes [*N* (successful trials) = 77, *N* (unsuccessful trails) = 15]. Subjects that were later successful in T3b (*N* = 6) tried this task (T3a) on average 87.5 ± 40.09 times unsuccessfully. In the last task 3b, seven individuals were able to extract food out of the boxes (mean number of successful trials = 131.14 ± 92.2). Here, individuals opened the boxes by applying two different techniques: two subjects opened the feeding boxes preferentially in the “correct” way (as described in the methods; T3) and raised the wooden board to the right from outside of the box opening. Five subjects found another way and slid the wooden board inside the box opening to the right (in the following referred to as “alternative” technique; see video Online Resource 4). Among the two subjects that opened the boxes with the “correct” technique, one individual also discovered the “alternative” technique but preferentially used the “correct” technique. Of the five subjects that opened the boxes with the “alternative” technique, two subjects also discovered the “correct” technique but used the “alternative” technique more often (Online Resource 5). In this task, subjects tried 28.71 ± 17.42 times unsuccessfully to obtain access to the reward before their first success.

Overall, the number of successful individuals varied between tasks (Cochrans *Q* test: T1–T2–T3: *N* = 28, *Q* = 12.286, *df* = 2, *P* = 0.002). Pairwise comparisons revealed that the number of individuals that succeeded did not vary between task 1 and 2, but between task 2 and 3 with fewer individuals being able to open the boxes in task 3 (McNemar test, T1–T2: *N* = 28, *P* = 1, T2–T3: *N* = 28, *P* = 0.031).

### Differences between tasks in problem-solving abilities and innovation

**Fig. 3:**
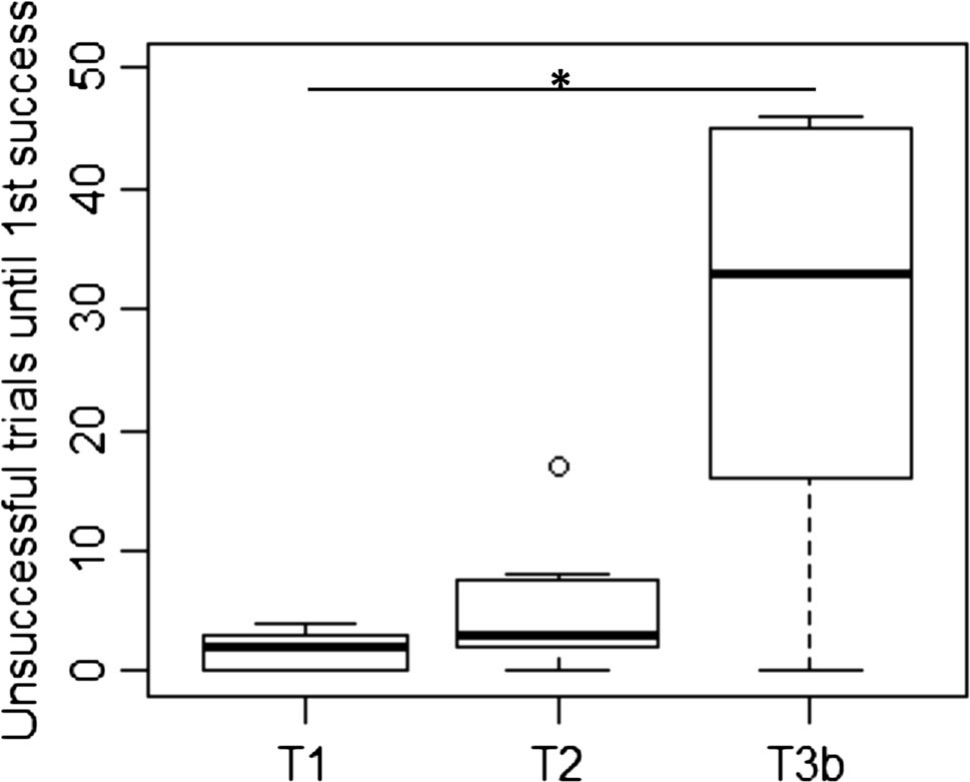
Number of unsuccessful trials until the first success in all three tasks (*N* = 7). Represented are median (*black bars*), interquartile range (*boxes*), upper and lower hinge (*whiskers*) and outliers (*circles*). *Asterisks* indicate significant differences with *P < 0.05

Among successful subjects, the number of unsuccessful trials performed before the first successful opening differed significantly between tasks (Friedman rank sum test, *N* = 7, *Q* = 7, *P* = 0.03). However, pairwise comparisons between tasks revealed that there was no difference in the number of unsuccessful trials between T1 and T2 (Wilcoxon matched pairs test, *N* = 14, *T* = 23.5, *P* = 0.422) as well as between T2 and T3b (*N* = 7, *T* = 3, *P* = 0.075) but between T1 and T3b (*N* = 7, *T* = 3, *P* = 0.036; Fig. 3).

The efficiency of successful subjects differed in the three different tasks significantly (Friedman rank sum test, *N* = 7, *Q* = 12.29, *P* = 0.002). Pairwise comparisons of efficiency between tasks revealed that individuals were more efficient in task 2 than in task 1 or in task 3 [Wilcoxon matched pairs test: T1–T2: *N* = 14, *T* = 17, *P* = 0.025, efficiency (mean ± SD): T1 = 0.72 ± 0.07, T2 = 0.81 ± 0.15; T2–T3b: *N* = 7, *T* = 28, *P* = 0.016, efficiency: T2 = 0.82 ± 0.09, T3b = 0.28 ± 0.16].

During the transition from task 1 to task 2, lemurs tried on average 2.36 ± 3.6 (SD) times unsuccessfully to open the boxes before their first success. Successful subjects applied on average 1.07 ± 1.44 times only the pull technique that was successful in task 1 before they began to further manipulate the boxes. In task 3, (a and b) successful subjects tried on average 10.28 ± 8.35 times to apply the previously rewarded technique of task 2 before their first successful trial.

Lemurs performed on average significantly more unsuccessful trials with the previous technique in task 3a (next task after task 2) than in task 3b [task 3a: 12.9 ± 13.98 (mean ± SD), task 3b: 2.15 ± 3.23, GLM, *P* < 0.001; Table 1]. There was also a significant interaction between session and the number of trials with the previous technique: While subjects applied the previous technique in the beginning of testing with task 3a and b extensively, this tendency decreased significantly across subsequent sessions (GLM, T3a: *P* < 0.001, T3b: *P* < 0.001; Table 1).

### How do successful subjects in task 3 differ from unsuccessful ones?

**Fig. 4:**
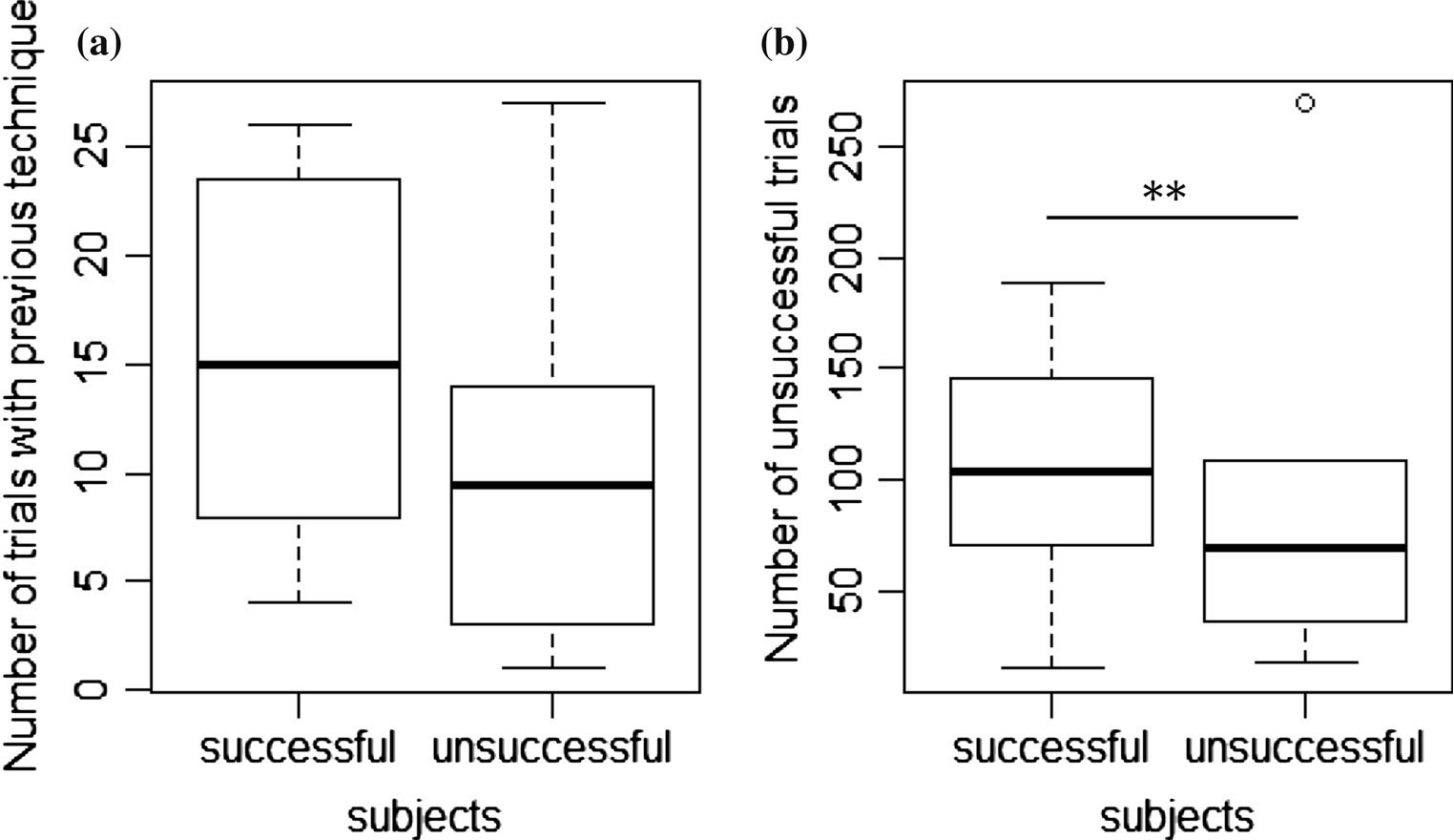
Comparison of successful subjects in task 3 (*N* = 7) and subjects that solved task 1 and 2 but failed to solve task 3 (*N* = 6) with respect to **a** the number of trials in which they applied the previously successful technique and **b** the total number of unsuccessful task manipulations during task 3. Represented are median (*black bars*), interquartile range (*boxes*), upper and lower hinge (*whiskers*) and outliers (*circles*). *Asterisks* indicate significant differences with **P < 0.01

The number of attempts with the previous technique did not differ between subjects that did succeed in task 3 and subjects that did not succeed but solved task 1 and 2 (T3 total: Mann–Whitney *U* test: *N* = 13, *Z* = 14.5, *P* = 0.39, Fig. 4). However, the total number of unsuccessful task manipulations was significantly higher in subjects that mastered task 3 (T3 total: Mann–Whitney *U* test: *N* = 13, *Z* = 2, *P* = 0.005, Fig. 4). Thus, subjects that tried more often, even without success, were more likely to solve the task.

Successful individuals had a higher exploration diversity score for each task than unsuccessful individuals (Mann–Whitney *U* test: T1: *N* = 29, *Z* = 195, *P* < 0.001; T2: *N* = 29, *Z* = 203, *P* < 0.001; T3b: *N* = 28, *Z* = 126, *P* = 0.003). Successful subjects always had the highest possible exploration diversity score in each task. Finally, the exploration diversity scores of subjects that did not manage to open boxes in any task did not differ significantly between the three tasks even though different maximum scores were possible (Friedman rank sum test, T1–T2–T3 total: *N* = 14, *Q* = 4.73, *P* = 0.094).

## Discussion

This study revealed that wild redfronted lemurs are capable of multiple innovations when presented with a novel feeding task, offering three different solutions to extract a reward with only one solution being successful at a time. Thus, redfronted lemurs are able to adjust their behavior flexibly to task constraints and build on (task 2) or abandon (task 3) previously learned problem-solving strategies after solutions are no longer successful, demonstrating behavioral flexibility and inhibitory control. Interestingly, in the most difficult test condition, successful and unsuccessful subjects did not differ in their degrees of conservatism, but in the total number of unsuccessful trials and thus in their persistence to manipulate the boxes. Thus, not only behavioral flexibility but also persistence is a relevant feature during flexible innovations in these wild lemurs.

Also, a subject’s general interest in the novel object or task was an important prerequisite for solving the presented problems. In general, neophobia may hamper innovation (e.g., Webster and Lefebvre 2001; Day et al. 2003; Greenberg 2003; Auersperg et al. 2011; Overington et al. 2011). In this study, only two subjects showed high levels of neophobia and never approached the boxes. During the course of testing, the number of subjects that contacted the feeding boxes successively increased during task 1 sessions, suggesting that familiarity with the set-up reduced neophobia in the course of testing. As tasks became more difficult, the number of animals interacting with the boxes decreased, however. Since the lemurs participated voluntarily and were therefore able to avoid the “problem” altogether, the decreasing number of participating animals during task 1 to task 3 may indicate that some individuals had limited behavioral flexibility, remaining unsuccessful and becoming less motivated when tasks became more difficult.

### Influence of sex, age and social learning

In the current study, female and male subjects were equally likely to succeed in opening the boxes, i.e., to innovate, with a slight bias toward females. Previous studies on the acquisition of problem-solving techniques in lemurs showed that females were more likely to innovate (Kappeler 1987; Dean et al. 2011; Schnoell and Fichtel 2012). A significant lack of a sex difference during innovation could be found in chimpanzees (Hrubesch et al. 2009) and hyenas (*Crocuta crocuta*: Benson-Amram and Holekamp 2012), but other studies in haplorrhine primates suggested a male bias (Reader and Laland 2001), indicating that sex alone is a weak predictor of innovativeness.

In contrast, subjects’ age affected their innovativeness as only adult redfronted lemurs were able to solve the different tasks. Since innovations require experience and skills, they are more likely to be found in adult individuals (Reader and Laland 2001). Conversely, during a social learning experiment with the same study population, innovators (*N* = 4) were individuals of less than 2 years (Schnoell and Fichtel 2012). Moreover, anecdotal reports suggested that younger individuals were more likely to innovate (Kummer 1971; Kummer and Goodall 1985; Hauser 1988). In the current study, juvenile subjects were generally interested in the feeding boxes and contacted them frequently by licking and sitting on them. However, with few exceptions, they did not manipulate boxes and thus did not learn to open them. Because older subjects already had experience with artificial feeding boxes from earlier studies (Schnoell and Fichtel 2012; Schnoell et al. 2014), this experience might have facilitated the adults’ problem-solving abilities.

Since lemurs were tested as a group in the wild, social learning might have influenced individual success. Although redfronted lemurs were shown to be able to learn socially (Schnoell and Fichtel 2012), they did not seem to use social information to open the boxes in the current study. Still, a general interest in the boxes might have been facilitated by stimulus or local enhancement (reviewed in Hoppitt and Laland 2008), which was not further tested here.

### Influence of previous knowledge, persistence and exploration on innovation

The first task in our study represented the basic problem that was given to the subjects, and the technique lemurs learned here was necessary to extract food rewards in all three tasks. Task 1 and task 2 were learned by most of the subjects (68 and 64 % of adult subjects, respectively). The number of unsuccessful trials until first success as a measure of difficulty and effort needed to extract a food reward was low in both tasks, and also efficiency in extracting rewards was high in task 1 and even higher in task 2. Thus, successful subjects learned task 1 and task 2 equally easily and fast, even though task 2 was more difficult, as subjects had to use both hands and perform different hand movements with each hand to open the boxes. In task 2, subjects did not need to inhibit the previously learned solution but to extend it, i.e., they added the new technique that was now necessary. Therefore, in task 2, lemurs presumably benefited from previous knowledge and experience and were able to learn the task quickly (see also Köhler 1925; Epstein et al. 1984; Kummer and Goodall 1985; Russon et al. 2010). Similarly, only individuals that had some experience with a certain technique were able to invent a cognitively more demanding but related technique in apes (Manrique et al. 2013). This flexibility in problem solving may result from a potential cumulative buildup of technology during individual learning (Lehner et al. 2011; Manrique et al. 2013).

In contrast, task 3 required a new problem-solving strategy at the same feeding boxes, i.e., a novel solution to an old problem. To solve this problem, subjects had to inhibit and abandon the previous technique in favor of a new and different technique. The difficulty of inhibiting a learned technique and learning a new one was reflected by the significantly smaller number of animals that solved this task. Moreover, subjects were less efficient in retrieving the rewards in this task than in task 2 and needed significantly more unsuccessful trials until they solved task 3 for the first time. However, the lemurs’ difficulties also reflect the generally more complex method required here; in the third task, the animals had to raise the wooden board from outside of the food entrance and could not simply follow the smell of the rewards during manipulation, presumably hampering trial-and-error learning.

Successful, flexibly innovating subjects in task 3 performed significantly more unsuccessful manipulations than subjects that failed to solve task 3. Even though subjects were not rewarded during all these unsuccessful attempts, they continued trying, demonstrating high degrees of persistence, despite occasional signs of frustration like biting the boxes. This biting could only be observed during unsuccessful manipulations of task 3 in 5 out of the 7 successful subjects. Persistence was already shown to influence innovation and problem-solving abilities in birds and other mammals [e.g., great tits (*Parus major*): Cole and Quinn 2012, keas: Gajdon et al. 2006, Carib grackles (*Quiscalus lugubris*): Overington et al. 2011, meerkats (*Suricata suricatta*): Thornton and Samson 2012, spotted hyenas: Benson-Amram and Holekamp 2012] and was found to be a consistent personality trait in chimpanzees (Massen et al. 2013).

In contrast, extreme forms of persistence, i.e., perseveration as a result of inhibitory problems (Hauser 1999), can also harm an animal’s ability to solve a problem, which often is the case when a new solution to an old problem must be found. Here, conservatism, reflected in perseverative errors subjects perform when repeating the same action over and over despite not being rewarded, prevents subjects from finding a novel solution. Animals stick to the solution they have initially learned even though an alternative technique might be more efficient. In chimpanzees, for example, subjects had difficulties in learning a second, more efficient technique to extract a food reward after they successfully learned a first one (Marshall-Pescini and Whiten 2008; Hrubesch et al. 2009; Gruber et al. 2011). In the study of Hrubesch et al. (2009), all three male chimpanzees that became specialists in a technique continued using this first technique even after it was made ineffective. In contrast, out of 14 redfronted lemurs that learned the technique required for task 2, seven were able to learn the successful technique in task 3. Thus, mastering a skill did not inhibit lemurs from learning a new technique, even though subjects exhibited perseverative errors in the beginning.

In great apes that had to invent a new strategy when a previously successful technique became ineffective, individuals that discovered the third, most difficult technique performed fewer errors with the previous technique than individuals that did not discover it, suggesting that individual conservatism may inhibit discovery and mastery of a skill in unsuccessful individuals (Manrique et al. 2013). However, in redfronted lemurs, successful and unsuccessful individuals did not differ in the number of perseverative errors. Although successful lemurs were conservative in the beginning of a new task, they succeeded in overcoming the predisposition for the old technique by being more persistent. Our findings therefore support the notion that not only behavioral flexibility plays a major role when finding a new solution to an old problem, but that also persistence and motivation are important prerequisites for success.

Interestingly, in task 3, successful subjects found two different ways to extract food rewards, with the majority of subjects applying efficiently the “alternative” technique, which was more similar to the previously learned technique in task 2. When applying the “alternative” technique, subjects manipulated the same parts of the box as in task 1 and 2 and basically applied the same technique as in task 2 (sliding) but in the other direction. Learning this new technique was therefore probably easier and required less behavioral flexibility. However, subjects had to exert much more strength to open the boxes with this technique compared with the “correct” technique, as no handle was available to open the boxes at this position. Thus, subjects that discovered this technique must have been very persistent and nimble. However, four subjects also applied the “correct” technique and were able to invent a totally new method to open the boxes.

Subjects also varied in the way they contacted the boxes, as reflected by the exploration scores of successful and unsuccessful individuals. Exploration scores of unsuccessful individuals were lower in all three tasks. Whereas unsuccessful individuals did not further explore the boxes, successful individuals had the highest possible exploration scores, i.e., they fully explored the boxes even though this was not mandatory to open them successfully. Thus, as in other species, lemurs’ explorative behavior appears to correlate with innovation and problem-solving abilities (Cole et al. 2011; Overington et al. 2011; Benson-Amram and Holekamp 2012).

## Conclusions

This study revealed that not only captive great apes, keas and New Caledonian crows, but also wild redfronted lemurs are able to innovate flexibly during problem solving when task conditions change and previously learned solution become obsolete. Besides behavioral flexibility, persistence, i.e., constant trying, was important for individual success during innovation. Thus, even phylogenetically basal primates are able to innovate flexibly, suggesting a general ecological relevance of behavioral flexibility and persistence during innovation and problem solving across all primates.

## References

[CR1] Anderson JR, Fornasieri I, Ludes E, Roeder JJ (1992). Social process and innovative behavior in changing
groups of Lemur fulvus. Behav Proc.

[CR2] Auersperg AM, von Bayern AMP, Gajdon GK, Huber L, Kacelnik A (2011). Flexibility in problem solving and tool use of kea and
New Caledonian crows in a multi access box paradigm. PLoS One.

[CR3] Benson-Amram S, Holekamp KE (2012). Innovative problem solving by wild spotted
hyenas. Proc R Soc B.

[CR4] Benson-Amram S, Weldele ML, Holekamp KE (2012). A comparison of innovative problem-solving abilities
between wild and captive spotted hyaenas, *Crocuta
crocuta*. Anim Behav.

[CR5] Berger J, Swenson JE, Persson I-L (2001). Recolonizing carnivores and naive prey: conservation
lessons from Pleistocene extinctions. Science.

[CR6] Birch HG (1945). The relation of previous experience to insightful
problem-solving. J Comp Psychol.

[CR7] Biro D, Inoue-Nakamura N, Tonooka R, Yamakoshi G, Sousa C, Matsuzawa T (2003). Cultural innovation and transmission of tool use in
wild chimpanzees: evidence from field experiments. Anim Cogn.

[CR8] Bond AB, Kamil AC, Balda RP (2007). Serial reversal learning and the evolution of
behavioral flexibility in three species of North American corvids (*Gymnorhinus cyanocephalus, Nucifraga columbiana, Aphelocoma
californica*). J Comp Psychol.

[CR9] Boogert NJ, Monceau K, Lefebvre L (2010). A field test of behavioural flexibility in Zenaida
doves (*Zenaida aurita*). Behav Proc.

[CR10] Bouchard J, Goodyer W, Lefebvre L (2007). Social learning and innovation are positively
correlated in pigeons (*Columba
livia*). Anim Cogn.

[CR11] Brooke ML, Davies NB, Noble DG (1998). Rapid decline of host defences in response to reduced
cuckoo parasitism: behavioral flexibility of reed warblers in a changing
world. Proc R Soc Lond Ser B.

[CR12] Brosnan SF, Hopper LM (2014). Psychological limits on animal
innovation. Anim Behav.

[CR13] Cole EF, Quinn JL (2012). Personality and problem-solving performance explain
competitive ability in the wild. Proc R Soc B.

[CR14] Cole EF, Cram DL, Quinn JL (2011). Individual variation in spontaneous problem-solving
performance among wild great tits. Anim Behav.

[CR15] Day RL, Coe RL, Kendal JR, Laland KN (2003). Neophilia, innovation and social learning: a study of
intergeneric differences in callitrichid monkeys. Anim Behav.

[CR16] Dean LG, Hoppitt W, Laland KN, Kendal RL (2011). Sex ratio affects sex-specific innovation and learning
in captive ruffed lemurs (*Varecia variegata*
and *Varecia rubra*). Am J Primatol.

[CR17] Duncker K, Lees LS (1945). On problem-solving. Psychol Monogr.

[CR18] Epstein R (1987). The spontaneous interconnection of four repertoires of
behavior in a pigeon (*Columba
livia*). J Comp Psychol.

[CR19] Epstein R, Kirshnit C, Lanza R (1984). ‘Insight’ in the pigeon: antecedents and determinants
of an intelligent performance. Nature.

[CR20] Fichtel C, Kappeler PM, Kappeler PM, Silk J (2010). Human universals and primate symplesiomorphies:
establishing the lemur baseline. Mind the gap: tracing the origins of human universals.

[CR21] Fornasieri I, Anderson JR, Roeder JJ (1990). Responses to a novel food acquisition task in 3
species of lemurs. Behav Proc.

[CR22] Gajdon GK, Fijn N, Huber L (2004). Testing social learning in a wild mountain parrot, the
kea (*Nestor notabilis*). Learn Behav.

[CR23] Gajdon GK, Fijn N, Huber L (2006). Limited spread of innovation in a wild parrot, the kea
(*Nestor notabilis*). Anim Cogn.

[CR24] Genty E, Palmier C, Roeder JJ (2004). Learning to suppress responses to the larger of two
rewards in two species of lemurs, *Eulemur
fulvus* and *E. macaco*. Anim Behav.

[CR25] Glady Y, Genty E, Roeder JJ (2012). Brown lemurs (*Eulemur
fulvus*) can master the qualitative version of the reverse-reward
contingency. PLoS One.

[CR26] Greenberg R, Reader SM, Laland KN (2003). The role of neophobia and neophilia in the development
of innovative behaviour of birds. Animal innovation.

[CR27] Gruber T, Muller MN, Reynolds V, Wrangham R, Zuberbühler K (2011). Community-specific evaluation of tool affordances in
wild chimpanzees. Sci Rep.

[CR28] Hanus D, Mendes N, Tennie C, Call J (2011). Comparing the performances of apes (*Gorilla gorilla*, *Pan
troglodytes, Pongo pygmaeus*) and human children (*Homo sapiens*) in the floating peanut
task. PLoS One.

[CR29] Hauser MD, Byrne RW, Whiten A (1988). Invention and social transmission: new data from wild
vervet monkeys. Machiavellian intelligence: social expertise and the evolution of
intellect in monkeys, apes and humans.

[CR30] Hauser MD (1999). Perseveration, inhibition and the prefrontal cortex: a
new look. Curr Opin Neurobiol.

[CR32] Heinrich B, Bugnyar T (2005). Testing problem solving in ravens: string pulling to
reach food. Ethology.

[CR33] Hoppitt W, Laland KN (2008). Social processes influencing learning in animals: a
review of the evidence. Adv Stud Behav.

[CR34] Hrubesch C, Preuschoft S, van Schaik C (2009). Skill mastery inhibits adoption of observed
alternative solutions among chimpanzees (*Pan
troglodytes*). Anim Cogn.

[CR36] Kappeler PM (1987). The acquisition process of a novel behaviour pattern
in a group of ring-tailed lemurs (*Lemur
catta*). Primates.

[CR38] Kappeler PM, Fichtel C, Kappeler PM, Watts DP (2012). A 15-years perspective on the social organization and
life history of sifaka in Kirindy Forest. Long-term field studies of primates.

[CR39] Kappeler PM, Fichtel C (2012). Female reproductive competition in *Eulemur rufifrons*: evidence and reproductive
restraint in a plurally breeding Malagasy primate. Mol Ecol.

[CR40] Kappeler PM, Barrett L, Blumstein DT, Clutton-Brock TH (2013). Constraints and flexibility in mammalian social
behaviour: introduction and synthesis. Philos Trans R Soc B.

[CR41] Kendal RL, Custance D, Kendal JR, Vale G, Stoinski T, Rakotomalala NI, Rasaminanana H (2010). Evidence for social learning in wild lemurs (*Lemur catta*). Learn Behav.

[CR42] Köhler W (1925) The mentality of apes, 2nd edn. Harcourt Brace, New York. Translated from German by E. Winter

[CR43] Kummer H (1971). Primate societies: group techniques of ecological
adaptation.

[CR44] Kummer H, Goodall J (1985). Conditions of innovative behaviour in
primates. Philos Trans R Soc Lond B.

[CR45] Laland KN, Reader SM (1999). Foraging innovation in the guppy. Anim Behav.

[CR47] Laland KN, Odling-Smee FJ, Feldman MW (1996). The evolutionary consequences of niche construction: a
theoretical investigation using two-locus theory. J Evol Biol.

[CR48] Lefebvre L, Heyes C, Huber L (2000). Feeding innovations and their cultural transmission in
bird populations. The evolution of cognition.

[CR49] Lefebvre L, Whittle P, Lascaris E, Finkelstein A (1997). Feeding innovations and forebrain size in
birds. Anim Behav.

[CR50] Lefebvre L, Gaxiola A, Dawson S, Timmermans S, Rosza L, Abai P (1998). Feeding innovations and forebrain size in Australasian
birds. Behaviour.

[CR51] Lefebvre L, Reader SM, Sol D (2004). Brains, innovations and evolution in birds and
primates. Brain Behav Evol.

[CR52] Lehner SR, Burkart JM, van Schaik CP (2011). Can captive orangutans (*Pongo
pygmaeus abelii*) be coaxed into cumulative build-up of
techniques?. J Comp Psychol.

[CR53] Lewis RJ (2002). Beyond dominance: the importance of
leverage. Q Rev Biol.

[CR55] Liker A, Bókony V (2009). Larger groups are more successful in innovative
problem solving in house sparrows. PNAS.

[CR58] Manrique HM, Völter CJ, Call J (2013). Repeated innovation in great apes. Anim Behav.

[CR60] Marshall-Pescini S, Whiten A (2008). Chimpanzees (*Pan
troglodytes*) and the question of cumulative culture: an
experimental approach. Anim Cogn.

[CR61] Massen JM, Antonides A, Arnold A-MK, Bionda T, Koski SE (2013). A behavioral view on chimpanzee personality:
exploration tendency, persistence, boldness, and tool-orientation measured with
group experiments. Am J Primatol.

[CR62] Morand-Ferron J, Quinn JL (2011). Larger groups of passerines are more efficient problem
solvers in the wild. PNAS.

[CR63] Morand-Ferron J, Cole EF, Rawles JEC, Quinn JL (2011). Who are the innovators? A field experiment with 2
passerine species. Behav Ecol.

[CR64] Nicolakakis N, Sol D, Lefebvre L (2003). Behavioural flexibility predicts species richness in
birds, but not extinction risk. Anim Behav.

[CR66] Overington SE, Cauchard L, Côté K-A, Lefebvre L (2011). Innovative foraging behaviour in birds: what
characterizes an innovator?. Behav Proc.

[CR71] Ramsey G, Bastian ML, van Schaik C (2007). Animal innovation defined and
operationalized. Behav Brain Sci.

[CR72] Reader SM, Biro D (2010). Experimental identification of social learning in wild
animals. Learn Behav.

[CR73] Reader SM, Laland KN (2001). Primate innovation: sex, age and social rank
differences. Int J Primatol.

[CR74] Reader SM, Laland KN (2002). Social intelligence, innovation, and enhanced brain
size in primates. PNAS.

[CR75] Reader SM, Laland KN (2003). Animal innovation.

[CR76] Reader SM, Hager Y, Laland KN (2011). The evolution of primate general and cultural
intelligence. Philos Trans R Soc Lond B.

[CR78] Russon AE, Kuncoro AE, Ferisa A, Handayani DP (2010). How orangutans (*Pongo
pygmaeus*) innovate for water. J Comp Psychol.

[CR80] Schnoell AV, Fichtel C (2012). Wild redfronted lemurs (*Eulemur rufifrons*) use social information to learn new foraging
techniques. Anim Cogn.

[CR81] Schnoell AV, Fichtel C (2013). A novel feeding behaviour in wild redfronted lemurs
(*Eulemur rufifrons*): depletion of spider
nests. Primates.

[CR82] Schnoell AV, Dittmann MT, Fichtel C (2014). Human-introduced long-term traditions in wild
redfronted lemurs?. Anim Cogn.

[CR84] Snell-Rood EC (2013). An overview of the evolutionary causes and
consequences of behavioral plasticity. Anim Behav.

[CR85] Sol D, Duncan RP, Blackburn TM, Cassey P, Lefebvre L (2005). Big brains, enhanced cognition, and response of birds
to novel environments. PNAS.

[CR87] Tebbich S, Fessl B, Blomqvist D (2009). Exploration and ecology in Darwin’s
finches. Evol Ecol.

[CR88] Tebbich S, Sterelny K, Teschke I (2010). The tale of the finch: adaptive radiation and
behavioural flexibility. Philos Trans R Soc Lond B.

[CR89] Thornton A, Samson J (2012). Innovative problem solving in wild
meerkats. Anim Behav.

[CR90] Timmermans S, Lefebvre L, Boire D, Basu P (2000). Relative size of the hyperstriatum ventral is the best
predictor of feeding innovation rate in birds. Brain Behav Evol.

[CR91] Turner SE, Fedigan LM, Matthews D, Nakamichi M (2012). Disability, compensatory behavior, and innovation in
free-ranging adult female japanese Macaques (*Macaca
Fuscata*). Am J Primatol.

[CR93] van Schaik CP, van Noordwijk MA, Wich SA (2006). Innovation in wild Bornean orangutans (*Pongo pygmaeus wurmbii*). Behaviour.

[CR94] Visalberghi E, Fragaszy DM, Savage-Rumbaugh S (1995). Performance in a tool-using task by common chimpanzees
(*Pan troglodytes*), bonobos (*Pan paniscus*), an orangutan (*Pongo pygmaeus*), and capuchin monkeys (*Cebus apella*). J Comp Psychol.

[CR95] Webster SJ, Lefebvre L (2001). Problem solving and neophobia in a
columbiform–passeriform assemblage in Barbados. Anim Behav.

